# Method development and validation for analysis of phenylalanine, 4‐hydroxyphenyllactic acid and 4‐hydroxyphenylpyruvic acid in serum and urine

**DOI:** 10.1002/jmd2.12287

**Published:** 2022-04-03

**Authors:** Andrew T. Hughes, Anna M. Milan, Ella Shweihdi, James Gallagher, Lakshminarayan Ranganath

**Affiliations:** ^1^ Department of Clinical Biochemistry and Metabolic Medicine Liverpool University Hospitals NHS Foundation Trusts Liverpool UK; ^2^ Bone and Joint Research Group, Musculoskeletal Biology The University of Liverpool Liverpool UK

**Keywords:** alkaptonuria, mass spectrometry, metabolites, nitisinone, tyrosine

## Abstract

Alkaptonuria (AKU) is a rare debilitating autosomal recessive disorder of tyrosine (TYR) metabolism which results in a deficiency of the enzyme homogentisate 1,2‐dioxygenase activity. Several studies have reported the metabolic changes in homogentisic acid (HGA) concentrations and subsequent deposition of an ochronotic pigment in connective tissues, especially cartilage. Treatment with nitisinone (NTBC) reduces urinary and circulating HGA, but its mode of action results in hypertyrosinaemia. The effect of NTBC on other metabolites in the TYR pathway has not been reported. Modification of the current reverse phase liquid chromatography tandem mass spectrometry methods for serum and urine to include phenylalanine (PHE), hydroxyphenyllactate (HPLA) and hydroxyphenylpyruvate (HPPA) has been validated. HPPA and HPLA (negative ionisation) eluted at 2.8 and 2.9 min respectively on an Atlantis C18 column with PHE (positive ionisation) eluting earlier at 2.4 min. Intra‐ and inter‐assay accuracy was between 96.3% and 100.3% for PHE; 96.6% and 110.5% for HPLA and 95.0% and 107.8% for HPPA in both urine and serum. Precision, both inter‐ and intra‐assay, was <10% for all analytes in both serum and urine. No significant issues with carry‐over, stability or matrix interferences were seen in either the urine or serum assays. Measurement of serum and urine from AKU patients has demonstrated a robust, fully validated assay, appropriate for monitoring of patients with AKU and for demonstrating metabolite changes, following NTBC therapy.

**Synopsis**

Measurement of intermediate tyrosine pathway metabolites will improve understanding of metabolic disease and its response to treatment.

## 1 INTRODUCTION

**FIGURE 1:**
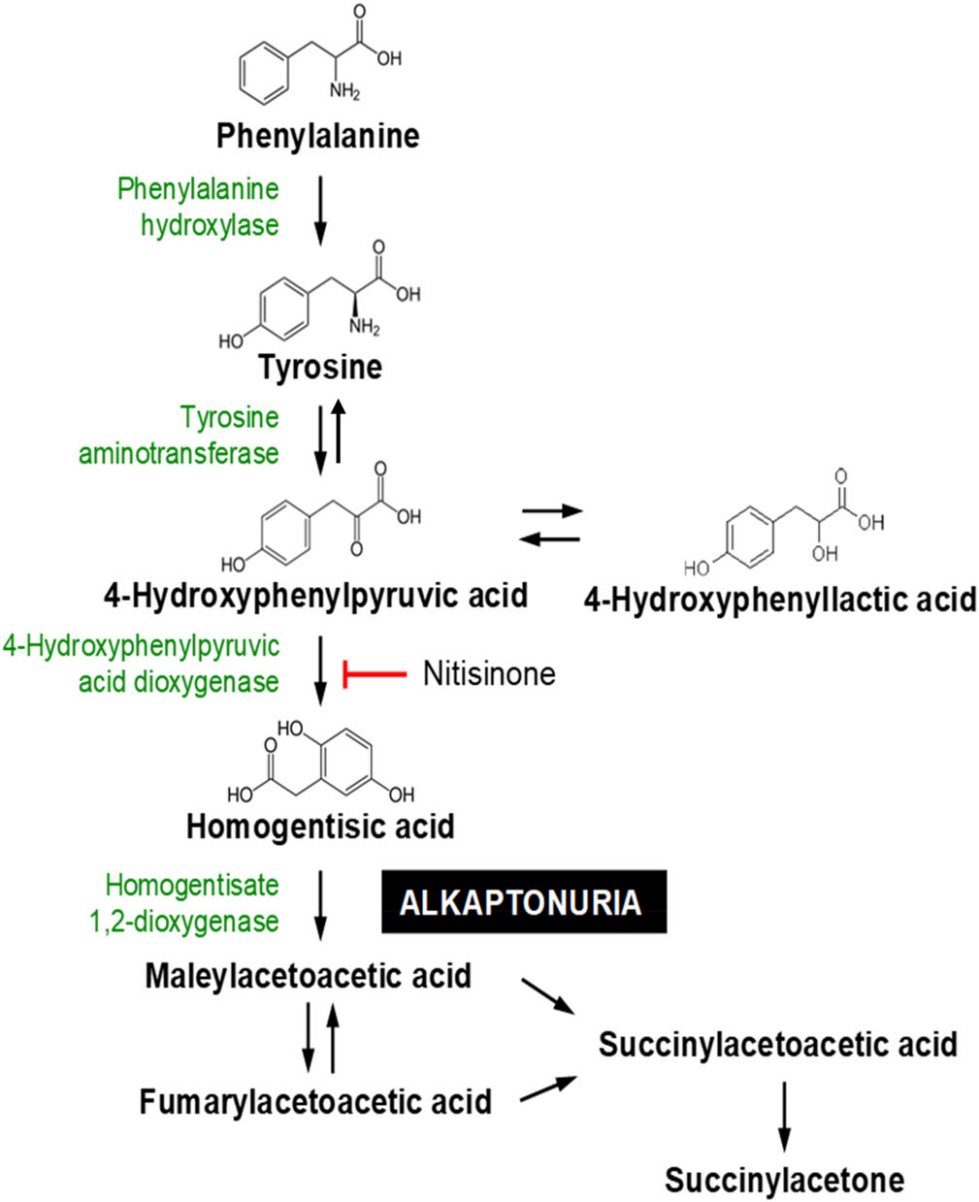
The tyrosine (TYR) pathway depicting the enzyme deficiency for alkaptonuria (AKU) and the enzyme blocking effect of nitisinone (NTBC)

Alkaptonuria (AKU) is a rare debilitating autosomal recessive disorder of tyrosine (TYR) catabolism. The TYR pathway along with its primary metabolites and enzymes has been well described^1^, ^2^, ^3^, ^4^ as displayed in Figure 1 and consists of a series of enzymatic reactions which yield acetoacetate and fumarate. Deficiency or severely reduced activity of these enzymes results in the autosomal recessive disorders commonly referred to as hereditary tyrosinaemia (HT) types 1, 2 and 3 and AKU. HT‐1, HT‐2 and HT‐3 are characterised by hypertyrosinaemia, however in AKU, normal plasma TYR concentrations are observed.^3^, ^5^, ^6^, ^7^ HT‐2 and HT‐3 are largely managed by a diet low in TYR and phenylalanine (PHE) to maintain TYR below a target of 500 μmol/L; whereas for HT‐1, nitisinone (NTBC) is an approved first‐line treatment.^8^, ^9^, ^10^ NTBC inhibits p‐hydroxyphenylpyruvate dioxygenase, the enzyme which leads to the formation of homogentisic acid (HGA), the compound that is the characteristic biochemical abnormality used in the diagnosis of AKU. It is also pathologically linked to the formation of the ochronotic pigment seen in AKU, although the exact mechanisms have not been fully elucidated.

NTBC has recently been approved for treatment of AKU^11^ and prior to this there was no approved disease modifying treatment for this disorder; analgesia and joint replacement being the main stays in the management of the musculoskeletal pain associated with AKU.^4^ Clinical trials into the use of NTBC for AKU have been on‐going and demonstrate the efficacy in reducing serum and urine HGA concentrations.^3^, ^5^, ^6^, ^7^, ^11^ The consequence, as in HT‐1, is hypertyrosinaemia.^3^, ^5^, ^6^, ^7^, ^11^, ^12^ Quantitative metabolite studies have largely focussed on measuring HGA and TYR; however, interest in the metabolites upstream has become interesting with regards to flux changes during treatment.^13^

The detection and measurement of hydroxyphenyllactate (HPLA) and hydroxyphenylpyruvate (HPPA) in the TYR pathway have been a challenge to analysts over the years largely due to their proposed instability and the reported undetectable levels in normal individuals. Previous methods for HPPA and HPLA have used paper chromatography,^14^ gas chromatography mass spectrometry with an extensive ether extraction protocol followed by sialylation,^15^ enrichment and isothermal capillary gas chromatography,^16^ high performance liquid chromatography (HPLC) with chemiluminescence^17^ and isotope dilution gas chromatography.^18^

This paper describes a validated liquid chromatography tandem mass spectrometry (LC–MS/MS) method for HPLA and HPPA with other key metabolites in the TYR pathway, namely PHE, HGA and TYR, in conjunction with serum NTBC.^1^, ^2^ This method is applied to three AKU patients to determine and demonstrate the changes in these metabolites in both serum and urine, upon commencing NTBC treatment.

## 2 MATERIALS AND METHODS

### 2.1 Chemicals and materials

The previously described methods for urine and serum TYR, HGA and NTBC (serum only) were modified to include a d_4_‐TYR internal standard (IS) (Hughes et al., 2014, 2015). TYR, PHE with their corresponding IS (d_4_ and d_5_ respectively), HGA, HPLA and HPPA were purchased from Sigma–Aldrich, UK. HGA isotope‐labelled IS ^13^C_6_‐HGA was obtained from Larodon Fine Chemicals (Sweden). LC–MS/MS grade methanol, acetonitrile (Honeywell, UK) and formic acid (Biosolve) were used throughout. Water was purified in‐house by the Elix Essential with a DIRECT‐Q 3UV Millipore water purification system. All dilutions and sample preparation were performed in glass. Oxygen‐free nitrogen was supplied by a Genius 3010 nitrogen generator from Peak Scientific.

### 2.2 Instrumentation and operating conditions

Analysis was performed on an Agilent 6490 Triple Quadrupole mass spectrometer with Jet‐Stream® electrospray ionisation (ESI–MS/MS) coupled with an Agilent 1290 Infinity UHPLC pump and 1290 multi‐sampler. All data processing both qualitative and quantitative analysis was performed using Mass Hunter software package (version B.06.00).

Chromatographic separation was achieved on an Atlantis dC18 column (100 mm × 3.0 mm, 3 μm, Waters) maintained at 35°C. Conditions were 20% organic (methanol, 0.1% formic acid) for 30 s, increasing linearly to 90% organic by 2.5 min, held for 1 min followed by 1.4 min at 100% organic and re‐equilibration to starting conditions for 2 min. Flow rate was 0.4 ml/min, and MS conditions were: gas temperature 150°C, gas flow 17 L/min, sheath gas temperature 320°C and sheath gas flow 12 L/min.

### 2.3 Preparation of standard solutions

Super‐stock standard solutions of HPPA and HPLA were made to a concentration of 400 mmol/L in deionised water (20–50 μl 40% sodium hydroxide for dissolution). PHE was dissolved at 10 mmol/L in deionised water (100 μl 5 N sulphuric acid for dissolution). All super‐stock solutions were sonicated prior to preparation of standards. Information regarding TYR, HGA and NTBC concentrations has been previously published.^1^, ^2^

### 2.4 Preparation of calibrators and controls urine and serum assay

To ensure a matrix‐matched calibration for the urine assay, super‐stock aqueous standard solutions were added to an acidified urine base pool (1% v/v 5 N sulphuric acid) which was assayed prior to preparation to verify minimal endogenous metabolites (due to stability of HGA all urine collections for AKU patients are collected into containers with 5 N sulphuric acid).

Initially, the super‐stock standards were diluted to intermediate stocks at five times the final required concentration. These were then added to the urine pool in a ratio of 1:4 (×5 dilution) to create combined calibrators with final concentrations ranging from 10 to 20 000 μmol/L for HPLA and HPPA and 5 to 250 μmol/L for PHE. In‐house quality controls (QC) were made in a similar manner independently covering the dynamic range of the calibration curve.

For the serum assay to ensure matrix‐matched calibration, super‐stock aqueous standard solutions were added to a serum matrix base pool (steroid depleted serum, BBI Solutions, SF236‐7) and again the base serum was assayed prior to preparation to verify minimal endogenous metabolites (TYR and PHE). The super‐stock standards were diluted to intermediate stocks at 10 times the final required concentration. These were then added to the steroid depleted serum matrix in a ratio of 1:9 (×10 dilution) to create combined calibrators with final concentrations ranging from 5 to 500 μmol/L for HPLA, 20 to 500 μmol/L for HPPA and 2 to 250 μmol/L for PHE. In‐house QC were made independently to cover the calibration range. TYR, HGA and NTBC were included at concentrations previously described.^1^, ^2^

### 2.5 Assay principle

A combined IS solution was used as the assay diluent, containing final concentrations of 500 nmol d_4_‐TYR, 12.5 nmol/L d_5_‐PHE and 1 μmol ^13^C_6_‐HGA per 500 ml deionised water with 0.1% formic acid for the urine assay and 250 nmol d_4_‐TYR, 12.5 nmol/L d_5_‐PHE and 100 nmol ^13^C_6_‐HGA and 1 nmol ^13^C_6_‐NTBC per 500 ml deionised water with 0.1% formic acid for the serum assay. All samples, calibrators and QC were assayed on a 1 in 1000 dilution with the above IS solutions.

### 2.6 Assay validation

The assay was validated using in‐house protocols based on published guidance.^19^, ^20^, ^21^ Only details pertaining to the validation of HPLA, HPPA and PHE are contained within this manuscript; for validation of TYR, HGA and NTBC readers are referenced to previous method publications.^1^, ^2^

#### 2.6.1 Linearity

Standard curves were fitted using linear regression with a 1/*x* weighting factor and a minimum of six calibration points plus matrix blanks (assayed first to check for endogenous levels) and curve‐fitting parameters excluded zero. Performance of fitted curves is presented as the coefficient of determination (*R* ^2^).

#### 2.6.2 Accuracy

Accuracy was determined as closeness to the nominal spiked concentrations, both intra‐ and inter‐assay with *n* = 6 and *n* = 20 respectively. Accuracy was calculated as: [measured concentration − nominal concentration]/[nominal concentration] × 100%.

No external quality assurance schemes exist for HPPA or HPLA. However for serum PHE, participation in ERNDIM (European Research Network for evaluation and improvement of screening, Diagnosis and treatment of Inherited disorders of Metabolism) was undertaken.

#### 2.6.3 Precision

Imprecision was determined both intra‐ (*n* = 6) and inter‐assay (*n* = 20) using separately spiked urine and serum pools and is expressed as coefficient of variation (%CV).

#### 2.6.4 Matrix effects

The presence of ion suppression was evaluated for HPPA, HPLA, PHE and their respective IS in both urine and serum matrices. Deionised water, acidified urine (three individual donors) were spiked with low, medium and high concentrations of HPPA, HPLA and PHE and each matrix was also spiked with the equivalent of the final concentration of IS. Similar was performed for deproteinised serum matrices. All spikes were individual analyte only.

Matrix factor was determined by calculating the ratio of the peak area in the presence of matrix (spiked with analyte or IS) to the peak area in the absence of matrix (deionised water plus analyte or IS). Using the matrix factors calculated, an IS normalised matrix factor can be determined (matrix factor of analyte/matrix factor of IS multiplied by 100).^19^ Noting in this case, ^13^C_6_‐HGA is also used as an IS for HPPA and HPLA (at the time of method development, no isotopic IS were available for HPLA and HPPA). More recently, isotopic IS for HPLA have been produced but this has not been implemented for continuity within the ongoing clinical trials.

#### 2.6.5 Dilution and carryover

Dilution integrity of urine and serum HPPA, HPLA and PHE was assessed by pre‐analytical dilution of five samples with high concentrations in deionised water at factors of 1 in 3, 5 and 10 with recovery as a percentage of the base sample analysed three times. Carryover of urine and serum HPPA, HPLA and PHE was assessed by five separate water injections following injection of the top calibrator.

#### 2.6.6 Stability

Stability of urine and serum HPPA, HPLA and PHE was assessed using three pools representing low, medium and high concentrations. Stability was determined following three freeze–thaw cycles (at −20°C), over 24 h at room temperature and over 24 h at 4°C (equivalent to the sample manager temperature, attached to the Agilent 6490). Results are expressed as a percentage of nominal values determined against a fresh calibration curve. Samples used for the on‐board 24 h stability were also repeatedly analysed over the 24 h period for any deterioration which may limit batch and run times.

### 2.7 Analysis of analytes in urine and serum samples

To confirm the utility of the assay, urine and serum from three patients, at three time points, were assayed from known AKU patients who attend the National AKU centre at Liverpool. Patients are administered a daily 2‐mg dose of NTBC and samples were analysed at baseline (pre‐NTBC), 6 months and 1 year. Analysis of these samples is covered under the NAC survey approved by the Institutional Audit Committee (Audit No: AC03836).

### 2.8 Analysis of analytes in healthy volunteer samples

Control samples were obtained following an amendment to the ethical application for the Natural History Study of AKU (NRES No 07/H1002/111). Participants (*n* = 22) were recruited from the Royal Liverpool and Broadgreen University Hospitals Trust and the University of Liverpool after obtaining informed written consent. The age range was 25–61 in females (*n* = 11) and 32–63 years in males (*n* = 11). None of the volunteers had AKU and all medications were documented.

## 3 RESULTS

### 3.1 Method validation

The mass spectrometer was operated in multiple reaction monitoring mode. Two product ion transitions were determined for each precursor ion and the respective collision energies are detailed in Table 1. For optimal sensitivity PHE and its IS were measured in positive ionisation mode while the acids HPPA, HPLA and ^13^C_6_‐HGA IS were measured in negative ionisation mode.

**TABLE 1:** Parameters for MS detection of HPPA, HPLA and PHE

Compound	Ionisation	Product ion (Quant) [CE]	Product ion (Qual) [CE]
PHE	Positive	166 > 91, [40]	166 > 77, [46]
HPLA	Negative	181 > 163, [10]	181 > 135, [14]
HPPA	Negative	179 > 134, [22]	179 > 107, [4]
d_5_‐PHE	Positive	171 > 125, [12]	171 > 106, [32]
^13^C_6_‐HGA	Negative	173 > 128, [24]	173 > 114, [20]

Abbreviations: HPLA, hydroxyphenyllactate; HPPA, hydroxyphenylpyruvate; MS, mass spectrometry; PHE, phenylalanine; CE, collision energy.

**FIGURE 2:**
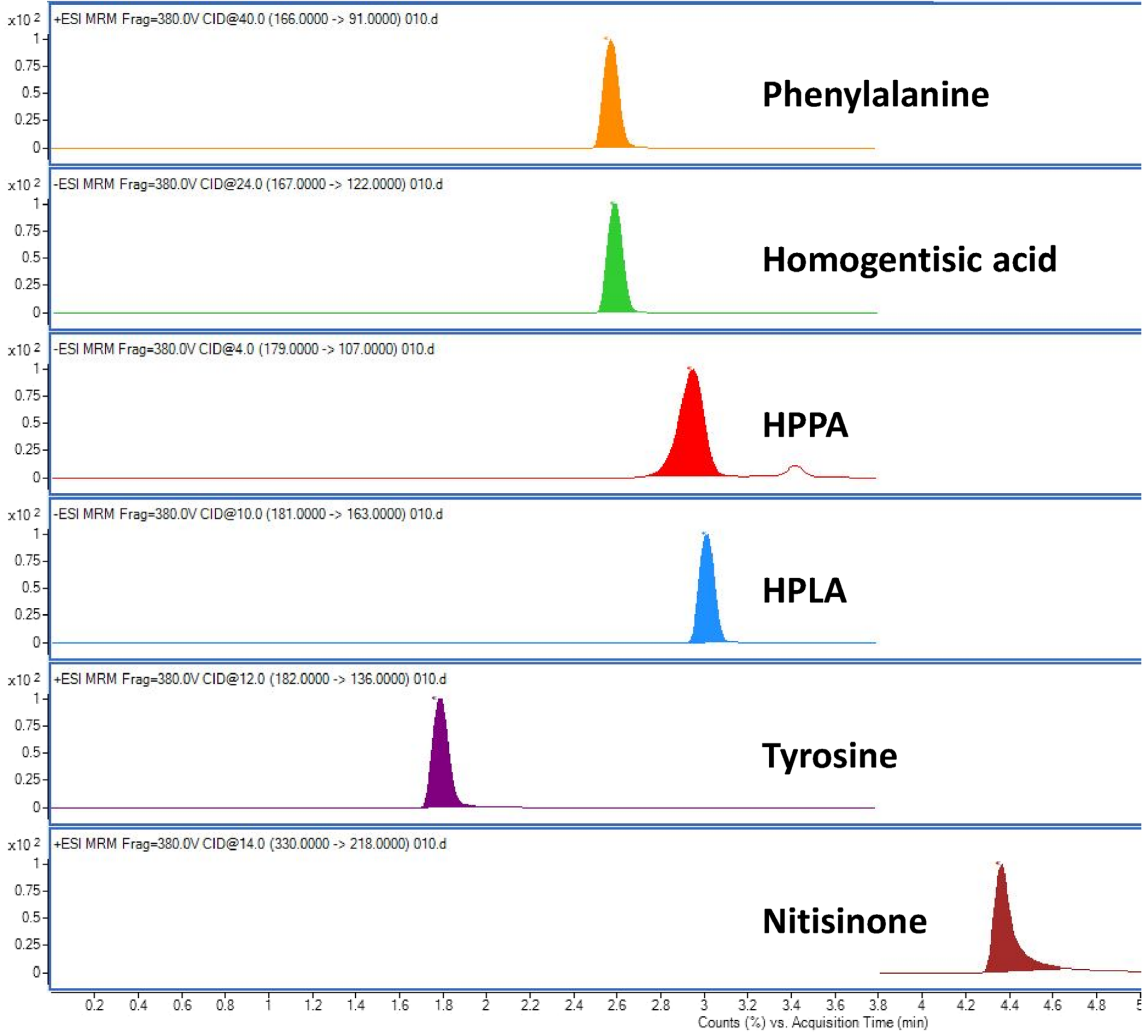
Chromatogram showing all quantifiable metabolites related to AKU in the TYR pathway (no IS included in view). This is a serum sample (PHE, TYR, HPPA and HPLA 250 μmol/L, HGA 25 μmol/L, NTBC 5 μmol/L). AKU, alkaptonuria; HGA, homogentisic acid; HPLA, hydroxyphenyllactate; HPPA, hydroxyphenylpyruvate; MS, mass spectrometry; NTBC, nitisinone; PHE, phenylalanine; TYR, tyrosine

A typical chromatogram is displayed in Figure 2 showing the full chromatographic separation of all measurable TYR pathway analytes (primary product ions or quantifiers only). Figure 2 is a serum sample (including NTBC). Chromatograms are identical for urine matrix (namely retention times) except NTBC is not quantified in the urine assay.

### 3.2 Linearity

Previous knowledge regarding the high urine HGA concentrations at baseline, in patients with AKU, and preliminary experiments on HPLA and HPPA, post‐NTBC, led to the understanding that urine concentrations would be in the same range as baseline HGA. Therefore, the measuring range validated in urine was considerably larger than that in serum. Although the assay is linear, the range of calibration standards chosen determined the measuring range stated in both urine and serum samples. For serum these are: PHE 10–525 μmol/L, HPLA 5–500 μmol/L, HPPA 10–540 μmol/L and urine: PHE 10–520 μmol/L, HPLA 20–20 000 μmol/L and HPPA 50–22 000 μmol/L.

Standard calibration curves (seven points including blank) exhibited a good fit over the range examined, with minimal inter‐assay variability over the concentration ranges described with *R* ^2^ = 0.999 for all analytes in both serum and urine (*n* = 8). For all assays, the calibration curve software was set at ‘exclude zero’ to ensure any contamination or baseline drift was detected.

### 3.3 Accuracy

Intra‐ and inter‐assay accuracy was determined in both urine and serum matrices (Table 2). Results are represented as percentage recovery of a nominal amount of HPPA, HPLA or PHE spiked into matrix. Intra‐ and inter‐batch accuracy was shown to be almost entirely within ±10% of the nominal expected value in both matrices.

**TABLE 2:** Intra‐ and inter‐assay accuracy for the urine and serum metabolite assays

Expected Conc (μmol/L)	Urine PHE		Expected Conc (μmol/L)	Urine HPLA		Expected Conc (μmol/L)	Urine HPPA	
	Intra	Inter		Intra	Inter		Intra	Inter
30	97.5 ± 3.4	97.7 ± 3.3	150	107.1 ± 4.5	109.4 ± 5.6	150	101.1 ± 4.1	103.6 ± 5.9
150	97.0 ± 3.1	97.7 ± 3.0	3000	110.3 ± 2.6	110.5 ± 4.1	3000	96.5 ± 5.8	95.0 ± 7.3
450	99.0 ± 3.7	99.4 ± 3.3	16 000	97.4 ± 3.1	96.6 ± 4.9	16 000	107.8 ± 4.2	101.7 ± 9.9

Expected Conc (μmol/L)	Serum PHE		Expected Conc (μmol/L)	Serum HPLA		Expected Conc (μmol/L)	Serum HPPA	
	Intra	Inter		Intra	Inter		Intra	Inter
20	96.3 ± 5.8	98.6 ± 5.6	25	108.5 ± 4.5	109 ± 5.1	70	100.9 ± 7.1	102.6 ± 9.5
150	99.7 ± 2.7	100.3 ± 3.0	150	100.5 ± 6.0	101.3 ± 5.0	150	101.9 ± 6.0	105.1 ± 7.5
450	97.4 ± 6.1	97.7 ± 5.3	450	103.7 ± 5.4	99.7 ± 6.4	450	103.7 ± 2.6	102.2 ± 4.6

Abbreviations: HPLA, hydroxyphenyllactate; HPPA, hydroxyphenylpyruvate; PHE, phenylalanine.

Participation in serum PHE EQA with ERNDIM has demonstrated acceptable performance for all samples (*n* = 8 per year) for the last 4 years to date.

### 3.4 Intra‐ and Inter‐assay precision

For intra‐assay, 10 aliquots of each analyte pool were analysed in a single batch with a calibration curve at the front and QC at beginning, middle and end of the run. For inter‐assay precision, the pools were assayed on 20 different runs across a 2‐week period. Assay validation requirements are for precision to be within a CV of 15% except for the lower limit of quantification (LLOQ) which should not exceed ±20%. All analytes performed within these criteria for both the urine and serum assay. For the urine assay: PHE <6% intra‐assay and <8.1% inter‐assay (10–520 μmol/L); HPLA <5% intra‐ and <9% inter‐assay (30–19 000 μmol/L) and HPPA <7.2% intra‐ and <10% inter‐assay precision (58–22 000 μmol/L).

For the serum assay: PHE <6.1% intra‐ and <5.6% inter‐assay (10–520 μmol/L); HPLA <10.9% intra‐ and <10.6% inter‐assay (6–506 μmol/L) and HPPA <11.0% intra‐ and <10% inter‐assay (30–533 μmol/L). It should be noted that precision was <7.5% for serum HPLA and HPPA if the lowest concentration pool was excluded from the average precision profile. No data have been trimmed and without exception, all assays have intra‐ and inter‐assay precision of acceptable performance.

### 3.5 Lower limit of quantification

The LLOQ is defined as the lowest calibrator which satisfies a CV ≤20%. This is generally considered the lowest calibration standard. The signal should also be at least five times the signal of a blank sample. The LLOQ (*n* = 10) for HPLA in serum was 5 μmol/L (CV 5.4%) and urine 20 μmol/L (%CV 7.5%); for HPPA in serum it was 10 μmol/L (CV 11.3%) and urine 50 μmol/L (CV 8.4%), and for PHE in serum it was 2 μmol/L (CV 5.2%) and urine 5 μmol/L (4.8%).

### 3.6 Matrix effect

The matrix effect of both serum and urine was assessed across the validated concentration range for HPPA, HPLA and PHE, with three individual urine and serum matrix pools. Results demonstrated that the IS counteracted any matrix suppression measured assuring acceptable levels of matrix effects normalised against the respective IS. For urine HPLA and HPPA there was a slight signal suppression (average −6.2% HPLA and −6.6% HPPA with CV <6.9% and <7.8% respectively); however, the IS normalised the suppression, behaving similarly, justifying the use of HGA IS for these two compounds. Serum HPLA and HPPA demonstrated a similar response with IS showing a normalised matrix factor 0.96–1.03 (HPLA) and 0.95–1.01 (HPPA) with %CV <10% for both across the concentrations examined. PHE in both serum and urine matrices satisfied validation criteria with CV <10% and minimal ion suppression. The matrix suppression in serum was high but as proven in the serum paper,^2^ the HGA IS behaves similarly leading to normalisation of the IS matrix factor.

### 3.7 Dilution integrity

Due to the large dynamic range of urine HPPA and HPLA concentrations exhibited post‐NTBC treatment, urine samples were tested at ×3, ×5 and ×10 dilution using deionised water, prior to assay preparation. Recovery percentages showed no effect on results when pre‐diluting samples, with both urine HPPA and HPLA with recovery at 1 in 10 of 96.5 ± 5.6 and 96.6 ± 6.6, respectively.

### 3.8 Stability

Stability following three freeze–thaw cycles demonstrated an average recovery of 97.3 ± 2.5% for urine HPLA, 96.8 ± 3.0% for urine HPPA and 98.1 ± 2.7% for urine PHE. For serum samples, similar recovery was demonstrated with 98.1 ± 3.4% for HPLA, 97.4 ± 3.3% for HPPA and 97.8 ± 2.1% for PHE. At room temperature, urine and serum samples showed no significant deterioration, although serum samples demonstrated a trend in decreasing concentration any samples kept at room temperature and not perchloric acid crashed within 12 h are not accepted for assay. Stability at 4°C demonstrated average recoveries 96.7 ± 4.5% for all analytes with repeat injections over a 24‐h period.

### 3.9 Carryover

Following injection of the top calibrator, there was no clear discernible peak in either HPPA, HPLA or PHE transition windows in the subsequent injected blank samples.

### 3.10 Crosstalk

HPPA and HPLA are structurally and chemically very similar. Prior to evaluation, it was essential to ensure that neither were detected within each other's analytical window and no conversion happened during the sample preparation and analytical process. Solutions of either HPPA or HPLA were injected periodically over a 24‐h period and both HPPA and HPLA areas were monitored. At no stage during this period was HPLA detected in the HPPA solution and vice versa. This period is unlikely to be exceeded during analysis time as a single assay batch can be assayed in less than 12 h.

### 3.11 Patient analysis

Serum and urine samples of three AKU patients treated with NTBC at the NAC were assayed using this validated method (Table 3). Routine measurement of TYR, HGA and NTBC (serum) was part of their scheduled review and the addition of HPPA, HPLA and PHE significantly enhances our understanding of the metabolic changes during treatment. At baseline, TYR and PHE are within reference range (3, 5–7), and a characteristically high serum and urine HGA is evident. HPLA and HPPA are undetectable at baseline (below LLOQ). Once NTBC treatment begins, the characteristic rise in TYR is seen in serum. Suppression of serum and urine HGA is observed, although it does remain detectable,^3^, ^7^ followed by a concurrent rise in HPLA and HPPA both in circulation and excreted through the kidneys.

**TABLE 3:** Tyrosine pathway metabolites in three AKU patients on NTBC (2 mg daily)

Patient	AKU serum	TYR (μmol/L)	PHE (μmol/L)	HGA (μmol/L)	HPLA (μmol/L)	HPPA (μmol/L)	NTBC (μmol/L)
1	Baseline	57	54	22.2	<5	<10	<0.2
	3 months	583	62	4.9	44	28	0.5
	1 year	793	67	4.8	37	32	0.6
2	Baseline	66	60	42.5	<5	<10	<0.2
	3 months	748	49	8.1	38	31	0.3
	1 year	741	69	3.5	59	41	1.1
3	Baseline	80	67	19.9	<5	<10	<0.2
	3 months	772	57	6.3	65	46	0.3
	1 year	777	49	2.7	70	40	0.6

Patient	AKU urine	TYR (μmol/24 h)	PHE (μmol/24 h)	HGA (μmol/24 h)	HPLA (μmol/24 h)	HPPA (μmol/24 h)
1	Baseline	45	29	21 063	<20	<50
	3 months	675	37	284	12 073	12 355
	1 year	759	35	393	14 067	15 841
2	Baseline	310	158	59 310	<20	<50
	3 months	3088	129	8946	17 923	37 944
	1 year	1852	42	2441	13 533	22 731
3	Baseline	104	41	23 912	<20	<50
	3 months	1011	39	2055	13 673	11 960
	1 year	1048	64	976	10 998	10 126

Abbreviations: AKU, alkaptonuria; HGA, homogentisic acid; HPLA, hydroxyphenyllactate; HPPA, hydroxyphenylpyruvate; NTBC, nitisinone; PHE, phenylalanine; TYR, tyrosine.

### 3.12 Healthy subject analysis

Serum HPPA and HPLA in all subjects were below the LLOQ for the assay; <10 and <5 μmol/L respectively. Serum PHE was detectable in all subjects with a mean of 64.1 μmol/L and a reference range of 41–80 μmol/L (95% percentile). Urine HPPA and HPLA were again undetectable in all urine samples, with concentrations below the LLOQ (<20 μmol/L HPLA and <50 μmol/L HPPA). Urine PHE results were reportable for 20 of the 22 subjects, with two having concentrations <5 μmol/L. Of the remaining subjects, the mean was 55.4 μmol/24 h (adjusted for 24 h urine volume) and 95% reference range 32.0–127 μmol/24 h.

## 4 DISCUSSION

A method has been validated for the simultaneous quantitation of urine and serum PHE and the TYR metabolites HPPA and HPLA. To date, this is the first published method for quantitation of these by LC–MS/MS and certainly the first method that now encompasses the full range of compounds related to AKU diagnosis and treatment in the TYR pathway. These additional analytes have been incorporated into the existing published methods^1^, ^2^ to enable a single sample to be utilised.

The method was validated with an uncomplicated sample preparation separating the compounds of interest across a short 7‐min chromatographical run‐time. It has been demonstrated as sensitive and specific with favourable accuracy and precision performance satisfying key validation guidelines.^19^, ^20^, ^21^

This all‐encompassing method also has the advantage over previously cited GC methods^15^ in that it avoids long laborious extraction techniques using volatile and toxic materials in favour of a liquid dilute and inject method for a fast and robust analysis of all the major compounds in the TYR pathway that are affected by AKU.

It is well understood that AKU patients have high circulating levels of HGA and once commenced on NTBC treatment, the levels decrease. Data from applying this method has demonstrated the significant consequential rise of metabolites HPPA and HPLA especially in urine (data in this paper^13^). The calibration range was evaluated to account for this in the majority of patients on NTBC treatment. We have further enhanced an existing method for thorough analysis of clinical trial and routine samples to determine the efficacy and response to NTBC in the treatment of AKU.

Levels of HPLA and HPPA are largely undetectable in health, and in untreated AKU individuals as the pathway either goes to completion (in health) without any enzyme deficiencies or blocks, or HGA is the predominant metabolite (in AKU). Previously, Crawhall et al.^15^ detected significant HPLA levels by GC in children with HT‐1. Levels of HPPA proved more challenging possibly due to the ether extraction procedure involved or perhaps the alkalinity of the urine samples causing sample degradation and loss of efficacy. This method was further expanded, although by no means made any less labour intensive, by Deutsch^18^ using isotopically labelled IS and a derivatising agent to enable detection at levels found in normal individuals, followed by GC, obtaining cleaner chromatograms. This author also noted instability in these compounds of 10% per month for HPPA and 3% per month for HPLA over a 10‐month period at −20°C.

Due to stability issues and for sample preservation, assay validation within this report was only performed in acidified urine (1% 5 N sulphuric acid) and perchloric acid precipitated serum samples.^1^, ^2^ This is standard throughout routine and trial sample preparation within our specialist AKU centre. Therefore the instability issues seen by Deutsch^18^ were also considerably reduced and further improved by storing samples at −80°C.

Deutsch^18^ stated that concentrations in health for HPPA (0.38 μmol/L) were similar to that found in a previous study,^17^ 0.34 μmol/L using HPLC with chemiluminescence detection. This is below the current LLOQ for the assay described so has not been confirmed. We have demonstrated that there are no measurable concentrations of HPPA or HPLA in healthy controls, in either serum or urine. Although the LLOQ are higher than the studies reporting HPPA in health, there is little clinical need within the AKU population to suggest further assay refinement at this concentration range.

In AKU patients treated with the drug NTBC we detect levels of these metabolites at significant concentrations especially in urine (mmol/24 h concentrations) and as methods have become more sensitive and sophisticated we are able to observe the behaviour of these metabolites in the disease and treated state with more accuracy. This becomes advantageous to the clinician in that they can build a picture of response to treatment and any effects of metabolite accumulation on the patients overall biochemistry. However, the predominant target in AKU patients is to suppress circulating HGA and subsequently urine HGA; control of HPPA and HPLA prior to treatment is not a clinical issue.

The method described here provides an enhanced method for thorough analysis of clinical trial and routine samples to determine the efficacy and response to NTBC in the treatment of AKU and other disorders of the TYR pathway.

## CONFLICT OF INTEREST

Andrew T. Hughes, Anna M. Milan, Ella Shweihdi and James Gallagher declare no conflict of interest. Lakshminarayan Ranganath received fees for lectures and consultations from Swedish Orphan Biovitrum.

## AUTHOR CONTRIBUTIONS

Andrew T. Hughes undertook method development and validation and the metabolic analysis reported within the manuscript and wrote the first draft of the manuscript. Anna M. Milan was involved with data review and reviewing the manuscript. Ella Shweihdi supported matrix factor experiments. James Gallagher reviewed the final draft of the manuscript and Lakshminarayan Ranganath was clinical director of the National AKU Centre at the time the work was undertaken and reviewed the final draft of the manuscript.

## ETHICS STATEMENT

Analysis of samples from patients with AKU is covered under the NAC survey approved by the Institutional Audit Committee (Audit No: AC03836). Control samples were obtained following an amendment to the ethical application for the Natural History Study of AKU (NRES No 07/H1002/111) at the Royal Liverpool and Broadgreen University Hospitals.

## ANIMAL RIGHTS

This article does not contain any studies with animal subjects performed by any of the authors.

## Data Availability

This manuscript has no associated data.
